# The Anaerobic Product Ethanol Promotes Autophagy-Dependent Submergence Tolerance in *Arabidopsis*

**DOI:** 10.3390/ijms21197361

**Published:** 2020-10-05

**Authors:** Li-Bing Yuan, Liang Chen, Ning Zhai, Ying Zhou, Shan-Shan Zhao, Li-Li Shi, Shi Xiao, Lu-Jun Yu, Li-Juan Xie

**Affiliations:** 1 State Key Laboratory of Biocontrol and Guangdong Key Laboratory of Plant Resources, School of Life Sciences, Sun Yat-Sen University, Guangzhou 510275, China; yuanlb5@mail.sysu.edu.cn (L.-B.Y.); zhaining@cemps.ac.cn (N.Z.); zhouying25@mail.sysu.edu.cn (Y.Z.); zhaoshsh6@mail2.sysu.edu.cn (S.-S.Z.); shilli@mail2.sysu.edu.cn (L.-L.S.); xiaoshi3@mail.sysu.edu.cn (S.X.); 2 State Key Laboratory for Conservation and Utilization of Subtropical Agro-bioresources, College of Life Sciences, South China Agricultural University, Guangzhou 510640, China; chenliang@scau.edu.cn

**Keywords:** ADH1, autophagy, ethanol, hypoxia, submergence

## Abstract

In response to hypoxia under submergence, plants switch from aerobic respiration to anaerobic fermentation, which leads to the accumulation of the end product, ethanol. We previously reported that *Arabidopsis thaliana* autophagy-deficient mutants show increased sensitivity to ethanol treatment, indicating that ethanol is likely involved in regulating the autophagy-mediated hypoxia response. Here, using a transcriptomic analysis, we identified 3909 genes in Arabidopsis seedlings that were differentially expressed in response to ethanol treatment, including 2487 upregulated and 1422 downregulated genes. Ethanol treatment significantly upregulated genes involved in autophagy and the detoxification of reactive oxygen species. Using transgenic lines expressing *AUTOPHAGY-RELATED PROTEIN 8e* fused to green fluorescent protein (*GFP-ATG8e*), we confirmed that exogenous ethanol treatment promotes autophagosome formation in vivo. Phenotypic analysis showed that deletions in the alcohol dehydrogenase gene in *adh1* mutants result in attenuated submergence tolerance, decreased accumulation of ATG proteins, and diminished submergence-induced autophagosome formation. Compared to the submergence-tolerant Arabidopsis accession Columbia (Col-0), the submergence-intolerant accession Landsberg *erecta* (L*er*) displayed hypersensitivity to ethanol treatment; we linked these phenotypes to differences in the functions of ADH1 and the autophagy machinery between these accessions. Thus, ethanol promotes autophagy-mediated submergence tolerance in Arabidopsis.

## 1. Introduction

Due to global climate change, flooding events are increasingly occurring worldwide, with detrimental effects on plant distribution and crop yields in natural ecosystems [1,2]. During flooding events, roots in the soil become surrounded by water (waterlogging), and most or all of the aerial tissue can become submerged under water (partial submergence or complete submergence) [3,4]. In such environments, plants can encounter hypoxia/anoxia damage, as the dramatic decrease in gas diffusion in floodwaters limits the availability of oxygen for aerobic respiration. The limited availability of oxygen and carbon dioxide, which hinders respiration and photosynthesis, together with the rapidly diminished availability of light for photosynthesis, can lead to carbohydrate starvation and an energy crisis in submerged plants [5,6,7].

Plants have developed both common and species-specific responses and strategies to endure or avoid excess water, allowing them to better adapt to and survive submergence stress. Anatomical changes such as aerenchyma formation improve internal gas diffusion, and morphological changes such as hyponastic and elongation growth help plants acquire oxygen and light above the water surface [5,6]. Several metabolic adjustments occur in plants in response to submergence. Ethylene biosynthesis is rapidly activated by submergence; this gaseous hormone becomes trapped in the plant due to restricted underwater gas diffusion [8,9]. Subsequently, the convergence of ethylene signaling with oxygen sensing activates the expression of hypoxia-responsive genes and downstream events to help the plant respond to low-oxygen conditions [8,9]. In addition, reactive oxygen species (ROS) produced by the activation of NADPH oxidases in complex with other proteins are essential for plant survival under submergence stress. Under hypoxia, ROS act as signaling molecules that regulate the expression of ROS-related genes involved in plant tolerance to submergence stress [10,11].

During submergence, ATP and the energy supplied to plant cells are derived from anaerobic fermentation instead of aerobic respiration [6]. In response to hypoxia stress, plants activate two major fermentation pathways that use pyruvate metabolized from starch reserves through glycolysis as a starting substrate to generate energy: lactic acid fermentation and primary ethanolic fermentation [6,7,12]. During lactic acid fermentation, the rapid activation of lactate dehydrogenase (LDH) catalyzes the reduction of pyruvate to lactate. The pyruvate can be further metabolized to alanine by alanine aminotransferase (AlaAT). The primary ethanolic fermentation pathway branches off the main glycolytic pathway at pyruvate. In this fermentation pathway, pyruvate decarboxylase (PDC) converts pyruvate to acetaldehyde, which is reduced to ethanol with the concomitant oxidation of NADH to NAD^+^ by alcohol dehydrogenase (ADH) [6,12]. The increased production of ethanol is considered to be benign, as ethanol can rapidly diffuse out of cells, except at high concentrations, whereas the intermediate acetaldehyde is toxic to plant cells [6,13].

ADH has been the subject of numerous genetic studies in plants [14]. In addition to *ADH1* (alcohol dehydrogenase 1) expression being activated in response to hypoxia/submergence and its significant role in hypoxia/submergence tolerance, *ADH1* expression is induced in various plant species by cold [15,16,17,18], dehydration [18,19,20], salt stress [18,20], wounding [21], hydrogen peroxide treatment [22], the phytohormones abscisic acid (ABA) and ethylene [18,23,24,25], and pathogen infection [18]. *Arabidopsis thaliana* plants overexpressing *ADH1* showed improved tolerance to dehydration, cold, and salt stress as well as increased resistance to pathogen infection, at least partially through the altered regulation of multiple stress-related genes, the accumulation of soluble sugars, and callose deposition [18]. Loss of function of ADH1 leads to changes in the levels of metabolites, including soluble sugars and amino acids, and it disrupts the normal connections among metabolites, as revealed by the metabolic profiling of plants exposed to cold stress [17]. Ethanol, a product of ethanolic fermentation mediated by ADH, enhances membrane lipid fluidity, thus preventing the lipid degradation that occurs in plants during cold injury [26,27]. Treatment with exogenous ethanol increased tolerance to salinity and cold stress in both rice (*Oryza sativa*) and Arabidopsis [27,28].

We previously reported that autophagy-defective mutants with markedly reduced tolerance to submergence stress showed enhanced sensitivity to exogenous ethanol treatment [29]. In submerged plants, autophagy is activated by the accumulation of ROS, which are by-products of anaerobic respiration, thereby regulating the hypoxia response during submergence [29]. The existence of upstream positive and negative regulators of autophagy, Snf1-related protein kinase 1 (SnRK1) and target of rapamycin (TOR), respectively, implies that a balancing mechanism modulates autophagy in plants. In response to an energy crisis, the SnRK1 complex senses changes in energy levels and the TOR complex is inactivated, allowing the activation of autophagy and downstream signals that promote plant survival [30]. Moreover, hypoxia induces the expression of the SnRK1 catalytic subunit genes *KIN10* and *KIN11*, and *KIN10-OE* plants showed enhanced tolerance to submergence [29,31]. These findings highlight the essential role of SnRK1-mediated energy sensing during autophagy in plant responses to hypoxia [29]. However, the role of ethanol in the autophagy-mediated plant response to submergence has been elusive.

Here, we revealed that genes involved in the autophagy machinery and the detoxification of ROS were significantly upregulated in response to ethanol. Exogenous ethanol treatment promoted autophagosome formation in transgenic lines expressing *autophagy-related protein 8e* fused to green fluorescent protein (*GFP-ATG8e*) transgenic plants. Moreover, *adh1* mutants with deficiencies in ethanol production showed impaired tolerance to submergence and diminished submergence-induced autophagosome formation. These observations suggest that ethanol generated from anaerobic fermentation likely plays an essential role in facilitating autophagy-mediated submergence tolerance in Arabidopsis.

## 2. Results

### 2.1. Ethanol Activates the Expression of Stress-Responsive Transcription Factors

We previously reported that autophagy-defective Arabidopsis mutants display enhanced sensitivity to submergence and ethanol application [29]. Given that ethanol is the end product of hypoxia-induced anaerobic respiration, we hypothesized that ethanol generated by anaerobic fermentation likely functions in the regulation of autophagy-mediated hypoxia responses. To test this hypothesis, we performed a transcriptomic analysis to identify ethanol-responsive genes in Arabidopsis. We treated two-week-old wild-type Arabidopsis Columbia (Col-0) seedlings with 50 mM exogenous ethanol or water for 12 h and then subjected the seedlings to high-throughput Illumina sequencing. We obtained 50.8–56.7 million 125-bp clean pair-end reads, 72.6−73.4% of which were uniquely mapped to the annotated Arabidopsis TAIR genome, indicating that the samples were comparable.

**Figure 1:**
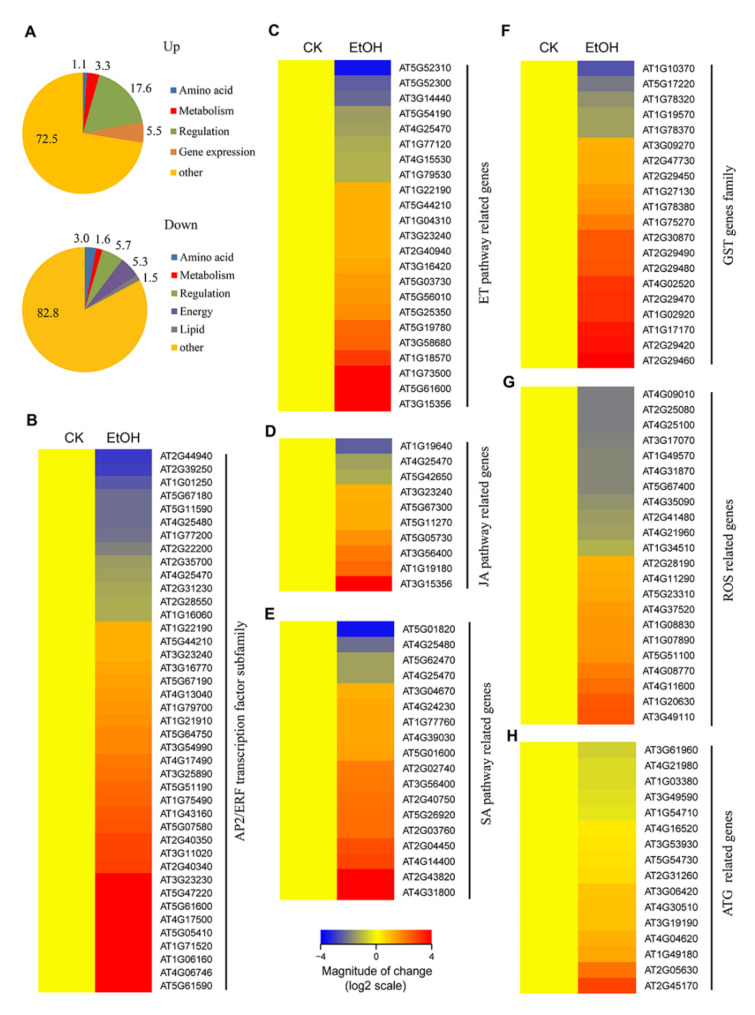
Differentially expressed genes in response to ethanol treatment. (**A**) Functional annotation of 2487 upregulated and 1422 downregulated genes after ethanol treatment. (**B**) Hierarchical clustering of differentially expressed ethanol-responsive genes from the AP2/ERF transcription factor subfamily. (**C**,**E**) Hierarchical clustering of differentially expressed ethanol-responsive genes from the ethylene (ET, **C**), jasmonic acid (JA, **D**), and salicylic acid (SA, **E**) biosynthesis and signaling pathways. (**F**,**H**) Hierarchical clustering of differentially expressed ethanol-responsive genes from the glutathione S-transferase (GST, **F**) gene family, reactive oxygen species (ROS, **G**)- and autophagy (ATG, **H**)-related genes. The log2 fold change values of the transcriptional profiles were calculated using the R program heatmap 2.0. Red and blue represent up- and downregulated genes, respectively. Differentially expressed genes were identified based on the criteria FDR < 0.005 and FC ≥ 1.5 or FC ≤ 0.67.

We identified 3909 differentially expressed genes (DEGs) that were significantly regulated by ethanol treatment (false discovery rate (FDR) < 0.005 and fold change (FC) ≥ 1.5), including 2487 that were induced and 1422 that were repressed by this treatment (Table S1, Figure S1A). Notably, ethanol treatment induced significant changes in the mRNA abundance of genes involved in regulation and energy, as indicated by functional annotation analysis (Figure 1A).

To further explore these DEGs, we performed GO and KEGG analyses using AgriGO [32], Metascape [33], and DAVID [34]. Among the upregulated genes, 18 GO terms were enriched (FDR < 0.05, Table S2), including eight GO terms related to abiotic stress (Figure S1A, Table S2). The GO terms “response to oxidative stress” (GO:0006979) and “response to salicylic acid” (GO:0009751) were overrepresented in the upregulated gene list (Table S2). Among the downregulated genes, eight GO terms were enriched (FDR < 0.05, Figure S1B, Table S2). GO terms associated with photosynthesis, including GO:0015979 and GO:0009768 (photosynthesis, light harvesting in photosystem I), were overrepresented in the downregulated gene list (Table S2). The KEGG pathways “ath00480: Glutathione metabolism” and “ath04075: Plant hormone signal transduction” were overrepresented among the 2487 upregulated genes (Table S3). The KEGG pathways “ath01110: Biosynthesis of secondary metabolites” and “ath00195: Photosynthesis” were overrepresented among the 1422 downregulated genes (Table S3). The KEGG pathway “ath04075: Plant hormone signal transduction” was also enriched among the 3909 DEGs (*P* < E−0.5) (Table S3).

Several transcription factor (TF) genes are differentially expressed during hypoxia/submergence, as revealed by global transcriptional profiling in Arabidopsis [35,36,37]. To identify TF genes that are differentially expressed in response to ethanol treatment, we examined the plant TF database PlantTFDB 4.0, which lists 2296 TF genes (1717 loci) in Arabidopsis that are classified into 58 families [38]. In our dataset, 274 TF genes (16.0%) were significantly differentially expressed, with 153 upregulated and 121 downregulated DEGs, which were grouped into 44 TF subfamilies (FDR < 0.005 and FC ≥ 1.5) (Table S4). Most DEGs belonged to the APETALA2/Ethylene-Response Factor (AP2/ERF), NAC, WRKY, bHLH, and MYB subfamilies (Figure 1B, and Figure S2). Indeed, AP2/ERFs TFs are known to play important roles in the submergence/hypoxia response [39].

We identified 41 differentially expressed AP2/ERF subfamily genes, including 13 that were downregulated and 28 that were upregulated in response to ethanol treatment (Figure 1B). Among these, *ERF107* (AT5G61590), *RAP2.9* (AT4G06746), and *ORA59* (AT1G06160) were induced 15.7-, 8.0-, and 7.5-fold, respectively, by ethanol (Figure 1B), suggesting that AP2/ERF TFs play important roles in the response to ethanol. In addition, 12 NAC and 14 WRKY TF genes were induced by ethanol treatment (Figure S2A,B), suggesting that NAC and WRKY TFs are likely responsible for activating ethanol-responsive genes. We also identified 27 differentially expressed bHLH subfamily genes, including 15 downregulated and 12 upregulated DEGs (Figure S2C). Moreover, we identified 40 differentially expressed MYB and MYB-related subfamily genes, including 22 downregulated and 18 upregulated DEGs. Among these, *RVE2* (AT5G37260) and *MYB90* (AT1G66390) were induced 31.5- and 7.6-fold, respectively, by ethanol treatment (Figure S2D). Finally, more than ten C2H2, CO-like, G2-like, and bZIP family genes and more than three TCP, Dof, C3H, HSF, DBB, GRAS, and Trihelix family genes were differentially expressed in response to ethanol (Table S4). Altogether, these data demonstrated that ethanol likely serves as a signaling molecule that regulates the expression of TF genes.

### 2.2. Expression Patterns of Phytohormone Pathway Genes in Response to Ethanol

To investigate the roles of phytohormones in the response of Arabidopsis to ethanol, we analyzed the expression of genes involved in phytohormone biosynthesis and signaling pathways. In total, 158 phytohormone pathway genes are differentially expressed in response to ethanol treatment (FDR < 0.005 and FC ≥ 1.5) based on the 694 Arabidopsis phytohormone-related genes in the AHD 2.0 database [40], including ABA, auxin, brassinosteroid (BR), cytokinin, ethylene, gibberellin (GA), jasmonic acid (JA), and salicylic acid (SA)-related genes.

Ethylene plays a key role in mediating the response to hypoxia/submergence in plants [8]. However, we did not identify any differentially expressed ethylene biosynthesis genes in response to ethanol treatment, indicating that ethanol does not induce ethylene accumulation. Among the ethylene response genes, *COR78* (AT5G52310) was downregulated 3.6-fold, while *TUA5* (AT5G19780) was upregulated 2.2-fold in response to ethanol treatment (Figure 1C). Among the ethylene signal transduction pathway genes, 10 of the 13 genes associated with signaling were induced by ethanol. Of these, *Lectin3.1* (AT3G15356, Legume lectin family protein), *ERF104* (AT5G61600), and *MKK9* (AT1G73500) were induced more than 4-fold in response to ethanol treatment (Figure 1C).

Six of the seven JA signaling pathway genes were upregulated by ethanol (Figure 1D). *TIFY10A*/*JAZ1* (AT1G19180), encoding a jasmonate ZIM-domain protein, was upregulated 2.2-fold upon ethanol treatment. In the SA pathway, *SGT1* (AT2G43820), encoding a nicotinate-O-glycosyltransferase in the SA metabolism pathway, was induced 3.9-fold by ethanol treatment. In addition, 12 of 16 SA signaling pathway genes were upregulated by ethanol, whereas no SA biosynthesis genes were differentially regulated by this treatment (Figure 1E), suggesting that the SA signaling pathway is also involved in the plant response to ethanol.

The expression levels of 43 genes involved in the ABA pathway were altered in response to ethanol, including four ABA biosynthesis genes, which were downregulated (Figure S3A). This result suggests that the ABA biosynthesis pathway is repressed by ethanol. In addition, seven ABA receptor (*PYR/PYL/RCAR*) genes, including *PYL1* (AT5G46790), *PYR1* (AT4G17870), *PYL7* (AT4G01026), *PYL4* (AT2G38310), *PYL6* (AT2G40330), *RCAR3* (AT5G53160), and *PYL5* (AT5G05440), were upregulated by ethanol treatment (Figure S3A), suggesting that ABA receptors are activated in response to ethanol. In the ABA signal transduction pathway, 13 genes were downregulated and 17 genes were upregulated (Figure S3A). Of these, *CCA1* (AT2G46830) was downregulated 46-fold and *BT2* (AT3G48360) was upregulated 27.9-fold in response to ethanol treatment (Figure S3A).

Among genes in other hormone pathways, *DRM2* (AT2G33830), encoding a dormancy/auxin-associated family protein, was induced 14-fold by ethanol compared to the control (Figure S3B). Four GA signal pathway genes were downregulated by ethanol treatment (Figure S3C). *ARR6* (AT5G62920), *ARR15* (AT1G74890), and *ARR9* (AT3G57040), encoding cytokinin response regulator proteins, were repressed by ethanol treatment (Figure S3D).

Fourteen genes in the BR biosynthesis and signaling pathways were differentially expressed in response to ethanol (Figure S3E). Among these, *DOGT1* (AT2G36800) was induced 7.8-fold by ethanol treatment; this gene encodes a DON-glucosyltransferase in the BR metabolism pathway involved in cellular responses to hypoxia. Finally, genes encoding the BR signaling components *EE3* (AT1G73830) and *EE1* (AT1G18400) were significantly upregulated by ethanol treatment (Figure S3E), suggesting that the BR pathway is also involved in plant responses to ethanol. Together, these results suggest a significant change in hormonal signal signaling in response to the ethanol treatment in Arabidopsis.

### 2.3. Ethanol Treatment Activates ROS Detoxification-Related Genes

ROS generated by NADPH oxidase are essential elements in the plant response to hypoxic stress, as they regulate the expression and activity of ADH1 [29,41,42,43]. The NADPH oxidase-deficient mutants *rbohd* and *rbohf* are hypersensitive to submergence stress and ethanol treatment [29,42]. Similar to submergence, ethanol treatment can trigger the generation of ROS [29,42]. We identified 42 DEGs associated with ROS in response to ethanol, including genes encoding glutathione reductase (GR), catalase (CAT), ascorbate peroxidase (APX), glutathione peroxidase (GPX), superoxide dismutase (SOD), glutathione S-transferase (GST), and peroxidase superfamily protein (POD) (Figure 1F,G). Among these, 15 of 20 GST genes were upregulated, including *GSTU3* (AT2G29470), *GSTF7* (AT1G02920), *GSTU24* (AT1G17170), *GSTU7* (AT2G29420), and *GSTU4* (AT2G29460), which were induced more than 3-fold by ethanol treatment (Figure 1F), demonstrating that the glutathione metabolic process is essential for the regulation of ethanol responses. In addition, four peroxidase superfamily protein (POD) genes (*PER39*, AT4G11290; *PER50*, AT4G37520; *PRX37*, AT4G08770; and *PRX33*, AT3G49110) were significantly upregulated by ethanol treatment (Figure 1G). Four of the five SOD DEGs (*CSD2*, AT2G28190; *FSD3*, AT5G23310; *CSD1*, AT1G08830; and *FSD2*, AT5G51100) were upregulated by ethanol (Figure 1G), and one GPX gene (*GPX6*, AT4G11600) and one CAT gene (*CAT1*, AT1G20630) were upregulated more than 2-fold in response to ethanol (Figure 1G). These results, combined with previous findings [28], indicate that exogenous ethanol activates genes encoding ROS scavengers, which helps the plant withstand increased ROS accumulation.

### 2.4. Ethanol Induces Autophagy

Autophagy-defective Arabidopsis mutants are hypersensitive to exogenous ethanol, exhibiting increased ROS accumulation [29]. In this context, the expression of 16 autophagy-related genes was altered by ethanol treatment, including two *ATG8* genes (*ATG8d* and *ATG8e*) that were induced more than 2-fold upon ethanol treatment (Figure 1H). These results suggest that exogenous ethanol regulates autophagy at the transcriptional level, which in turn affects the activation of autophagy.

**Figure 2:**
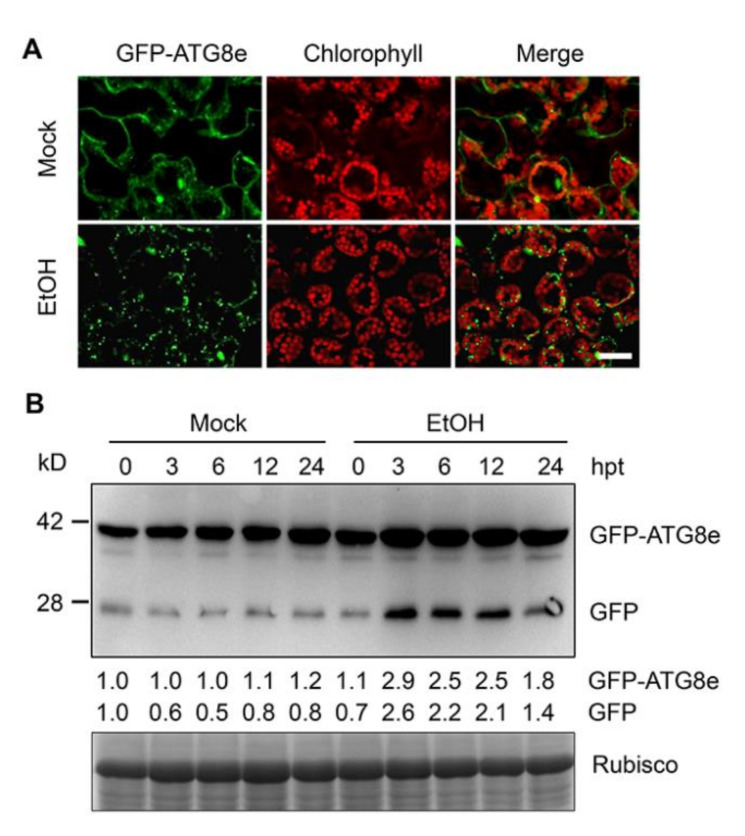
Induction of autophagy by ethanol treatment. (**A**) Confocal analysis of changes in transgenic lines expressing *autophagy-related protein 8e* fused to green fluorescent protein (GFP-ATG8e) in leaf cells in response to ethanol treatment. Leaves of 4-week-old *GFP-ATG8e* transformants were detached and immersed in 100 mM ethanol or water for 6 h, and GFP-ATG8e was visualized by fluorescence confocal microscopy. Bar, 50 µm. (**B**) Immunoblot analysis showing the free GFP generated from GFP-ATG8e fusion protein upon ethanol treatment. Detached leaves from 4-week-old *GFP-ATG8e* plants were immersed in 100 mM ethanol or water, and leaves were collected at 0, 3, 6, 12, and 24 h after treatment. Anti-GFP antibodies were used for immunoblotting. Coomassie blue-stained total proteins are shown at the bottom to indicate the amount of protein loaded per lane. Numbers below the protein bands indicate relative gray values of the bands. The numbers on the left indicate the molecular mass (kD) of the size markers. hpt, hours post-treatment.

To further confirm this hypothesis, we examined the formation of autophagosomes, which reflect the genesis and progress of autophagy, in leaf epidermal cells of *GFP-ATG8e* transgenic plants [44]. The formation of GFP-labeled punctuate autophagosomes was markedly induced upon 100 mM ethanol treatment (Figure 2A). To verify this observation, we analyzed the release of free GFP to detect the degradation of GFP-ATG8e, which is widely used to determine the rate of autophagy [29]. Consistent with the confocal microscopy observations, immunoblotting using anti-GFP antibody revealed that the levels of free GFP were markedly enhanced by ethanol treatment; this increase was maintained after 12 h of ethanol treatment (Figure 2B). Furthermore, the accumulation of GFP-ATG8e was induced by ethanol at all-time points (3, 6, 12, and 24 h of treatment) compared to mock conditions (Figure 2B). These observations, together with the results of transcriptomic analysis, strongly suggest that autophagy is induced in response to ethanol.

### 2.5. Disruption of ADH1 Attenuates Submergence-Induced Autophagy

ADH is a crucial enzyme that catalyzes the conversion of aldehyde to ethanol during ethanolic fermentation in plants under submergence stress [12,25]. To further explore the roles of ADH1 and endogenous ethanol in submergence-induced autophagy, we isolated three *adh1* null mutants harboring insertions/deletions in *ADH1* [45] (Figure S4A). Sequencing confirmed that *adh1-4* (CS66116) and *adh1-16* (CS66118) harbored base insertions and that *adh1-8* (CS66117) harbored deletions in *ADH1*, which resulted in errors in the coding sequences of Gly68 (*adh1-4* and *adh1-8*) or His69 (*adh1-16*) and disrupted the expression of *ADH1* (Figure S4A–C). Furthermore, previous studies revealed extremely low ADH activity in all *adh1* mutants [18,46,47]. When germinated on solid MS medium containing 50 mM ethanol, the *adh1* mutants showed increased tolerance to ethanol, with longer roots than the wild type (Figure S4D). Consistent with previous finding, *adh1* mutants exhibit increased tolerance to allyl alcohol [45,48]. It is possible that that when encountering stress conditions with a high accumulation of ethanol, ADH1 would contribute to the aerobic conversion of ethanol to toxic acetaldehyde [14,49]. By contrast, the loss of ADH1 function in *adh1* mutants would keep them surviving from long-term ethanol treatment [45,48].

**Figure 3:**
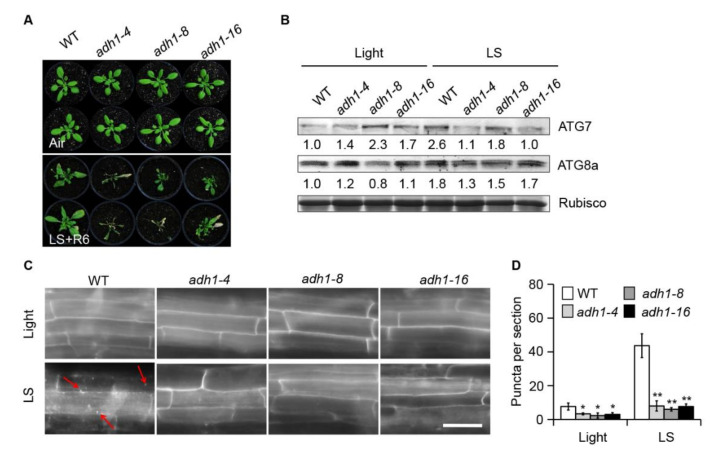
Loss of function of ADH1 attenuates submergence tolerance and autophagosome formation. (**A**) Phenotypes of 4-week-old wild-type (WT), *adh1-4*, *adh1-8*, and *adh1-16* plants before treatment (Air) and following 6 d of recovery after 7 d of light (normal light/dark cycle) submergence (LS) treatment (LS+R6). (**B**) ATG7 and ATG8a protein levels in 4-week-old WT, *adh1-4*, *adh1-8*, and *adh1-16* plants before treatment (Light) and after 24 h of LS treatment. Coomassie blue-stained total proteins are shown below the blots to indicate the amount of protein loaded per lane. (**C**) Monodansylcadaverine (MDC) staining of mature root cells from one-week-old WT, *adh1-4*, *adh1-8*, and *adh1-16* seedlings under normal light/dark (Light) conditions or following 24 h of LS treatment. Red arrows indicate labeled autophagosomes. Bars = 50 µm. (**D**) Number of puncta per root section in mature root cells from the WT, *adh1-4*, *adh1-8*, and *adh1-16* seedlings shown in (**C**). Data are average values ± SD of three biological replicates. For each experiment, 15 sections were analyzed per genotype. Asterisks represent significant differences between WT and *adh1* samples, as determined by Student’s *t*-test (* *p* < 0.05 and ** *p* < 0.01).

The general treatment in submergence research is under constant darkness, but it is also acceptable to do submergence experiments under normal light/dark cycles conditions [29,50,51]. Given that constant darkness may activate autophagy [29,44], we performed submergence treatment under normal light/dark cycle conditions (LS) in subsequent experiments related to autophagy. Consistent with previous findings [48,49,52], all three mutant alleles of *ADH1* displayed significant hypersensitivity to submergence compared to wild-type plants (Figure 3A). When germinated on solid MS medium containing 50 mM ethanol, the *adh1* mutants showed increased tolerance to ethanol, with longer roots than the wild type (Figure 3B). Consistent with this finding, *adh1* mutants exhibit increased tolerance to allyl alcohol [45,48].

To obtain further details about the roles of ADH1 and ethanol in regulating submergence-induced autophagy, we measured the abundance of the autophagy-related proteins ATG7 and ATG8a in the *adh1* mutants to assess the potential link between ADH1 and autophagy-mediated submergence responses. Whereas ATG7 levels increased in wild-type plants exposed to submergence for 24 h, loss-of-function mutations in *ADH1* led to reduced ATG7 levels (Figure 3B), suggesting that submergence-induced ATG7 accumulation is dependent on ADH activity. Analysis of ATG8a accumulation showed that ATG8a levels in Col-0 plants increased in response to submergence, whereas ATG8a levels were lower in *adh1* than in the wild type (Figure 3B). Accordingly, upon submergence treatment, the transcript levels of *ATG* genes were significantly lower in the *adh1* mutant than that of Col-0 plants (Figure S5). These results indicate that the submergence-induced accumulation of ATGs is dependent on ADH1. Moreover, monodansylcadaverine (MDC) staining of the root cells of wild-type and *adh1* seedlings indicated that ADH1 functions in autophagosome formation under submergence (Figure 3C). Submergence-induced autophagosomes were clearly visible in mature root cells of wild-type plants; however, autophagosome formation was blocked in the root cells of the three *adh1* mutants (Figure 3C). Analysis of the number of puncta per root section in mature root cells of wild-type and *adh1* plants confirmed that disrupting *ADH1* attenuates submergence-induced autophagosome formation (Figure 3D).

These results suggest that *ADH1*, encoding an enzyme that catalyzes the conversion of aldehyde to ethanol during ethanolic fermentation, is essential for submergence-induced autophagy.

### 2.6. The Submergence-Intolerant Arabidopsis Accession Shows Increased Sensitivity to Ethanol

We previously reported that some *Arabidopsis thaliana* genotypes show similar levels of tolerance to submergence and exogenous ethanol treatment and that treatment with ethanol (the end product of hypoxia-induced ethanolic fermentation) could be used to mimic hypoxic stress under certain conditions [29,50,51]. Arabidopsis *ADH1* overexpression lines (with increased ADH activity under hypoxic conditions in both roots and shoots) did not show improved submergence survival compared to the wild type [52], suggesting that these lines might not be the best experimental materials for subsequent experiments. We noticed that different Arabidopsis accessions show natural variation in submergence tolerance, providing a potential resource for identifying the molecular mechanisms underlying the plant response to submergence [53,54].

**Figure 4:**
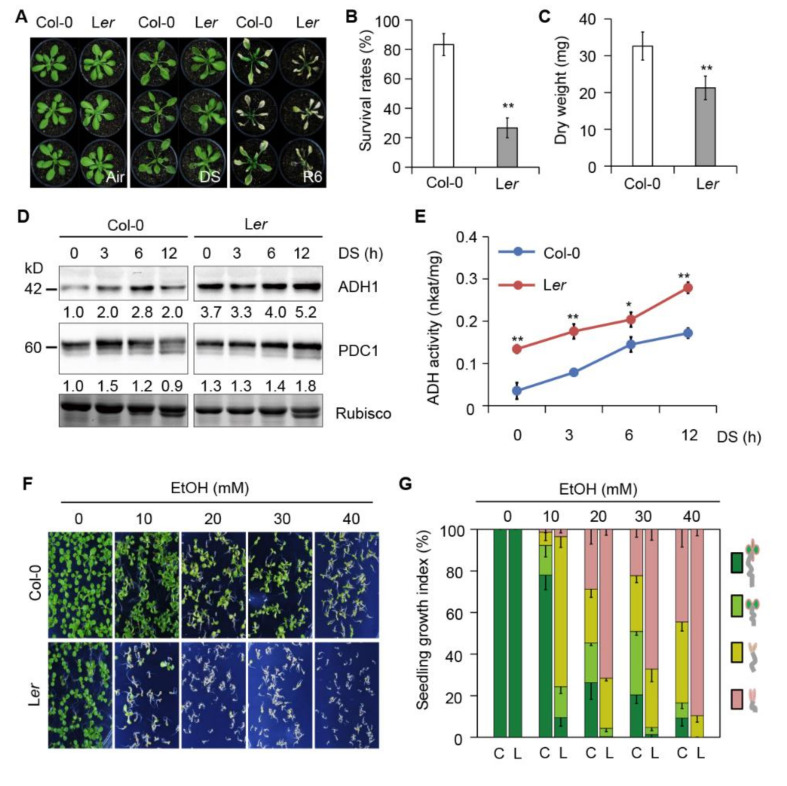
Two Arabidopsis accessions with different levels of submergence tolerance show different responses to ethanol. (**A**) Phenotypes of 4-week-old Columbia (Col-0) and Landsberg *erecta* (L*er*) plants before submergence (Air) and after 60 h of dark submergence (DS), followed by recovery for 6 d (R6). The experiments were repeated three times with similar results. (**B**) and (**C**) survival rate (**B**) and dry weights (**C**) of Col-0 and L*er* plants after DS treatment followed by recovery for 6 d. Data are means (± SD) of three biological replicates. For each biological repetition, 15 plants were used per accession. Asterisks indicate significant differences between Col-0 and L*er*, as determined by Student’s *t*-test (** *P* < 0.01). (**D**) Protein abundance of ADH1 and PDC1 in 4-week-old Col-0 and L*er* plants treated with dark submergence (DS) at various time points. Numbers below the protein bands indicate relative gray values of the bands. Coomassie blue-stained Rubisco is shown as a loading control. The experiments were repeated three times with similar results. (**E**) Measurement of alcohol dehydrogenase (ADH) activity in 2-week-old Col-0 and L*er* plants after dark submergence (DS) treatment for 0, 3, 6, and 12 h. The experiments were biologically repeated three times with similar results. Error bars represent SD (*n* = 3 technical replicates). Asterisks indicate significant difference between Col-0 and L*er*, as determined by Student’s *t*-test (* *p* < 0.05 and ** *p* < 0.01). (**F**) Col-0 and L*er* seedlings grown on MS medium supplemented with different concentrations of ethanol. Images were taken 10 d after germination. The experiments were repeated three times with similar results. (**G**) Seedling growth index in (**F**). The colors in the columns correspond to seedlings with true leaves (dark green), seedlings with green (light green) or brown (yellow) cotyledons, and etiolated seedlings (pink). Data are means (± SD) of three biological replicates. C, Col-0; L, L*er*.

To gain insight into the link between ethanol and submergence tolerance, we examined the submergence tolerance of the commonly used laboratory Arabidopsis accessions Col-0 and Landsberg *erecta* (L*er*), which previously showed obvious variations in tolerance submergence [53]. Four-week-old plants were completely submerged for 60 h in the dark, which was followed by a return to control growth conditions for 6 d of recovery (Figure 4A). Col-0 maintained more green leaves (Figure 4A), higher survival rates (Figure 4B), and greater shoot dry weight (Figure 4C) than the sensitive accession L*er* during the recovery period. As a control, when Col-0 and L*er* plants were placed in the dark with no submergence treatment, there were no significant phenotypic differences between plants during the dark treatment and recovery phases vs. control treatment for both accessions (Figure S6).

Plants switch to ethanolic fermentation, in which pyruvate is converted to ethanol through the coupled reactions of PDC and ADH, to cope with the energy crisis triggered by submergence [6,12]. In response to oxygen deprivation, higher PDC1 and ADH1 levels and activity are induced, thereby increasing the energy supply and ethanol production [6,12]. To explore whether the differences in the submergence tolerance of Col-0 and L*er* are associated with differences in the ethanolic fermentation pathway, we measured ADH1 and PDC1 abundance and ADH activity in both accessions. ADH1 protein levels increased in Col-0 after 3, 6, and 12 h of submergence treatment in the dark (Figure 4D). By contrast, before dark-submergence treatment (0 h), ADH1 protein levels were higher in L*er* than in Col-0, and they remained at higher levels during the course of treatment (Figure 4D). Unlike ADH1, in both accessions, PDC1 protein levels were relatively high and did not significantly change in response to treatment, despite being weakly enhanced in L*er* plants in response to submergence treatment in darkness (Figure 4D). The transcript levels of *ADH1* and *PDC1* did not significantly differ between the two accessions, although both were induced by submergence treatment (Figure S7A, B). Previous study revealed that amino acids are crucial for enzyme activity and dimer stability for *Drosophila* alcohol dehydrogenase [55,56,57]. Consistent with these reports, amino acid sequence alignments of ADH proteins form different species showed that amino acid differences between two accessions were observed near the conserved residues of the catalytic domain (Figure S7C). Accordingly, ADH enzyme activity was induced by submergence stress in both accessions, with L*er* showing higher ADH activity than Col-0 at all time points examined (Figure 4E). In addition, upon ethanol treatment, the endogenous ethanol accumulation was significantly higher in L*er* plants than that in Col-0 (Figure S7D). These results indicate that the submergence-intolerant accession L*er* possesses higher ADH1 protein abundance and activity during submergence than the submergence-tolerant accession Col-0. These results suggest that the ethanol produced by enhanced ethanolic fermentation may affect plant tolerance to submergence to some extent.

To confirm this hypothesis, we examined seed germination and post-germination growth in both accessions in response to exogenous ethanol. As shown in Figure 4F, when grown on solid MS medium, germination and post-germination growth were increasingly inhibited in Col-0 in response to increasing concentrations of ethanol. However, compared to Col-0, the submergence-sensitive accession L*er* was more sensitive to ethanol treatment. L*er* seedlings exhibited delayed germination and stagnated postgerminative growth under 10 mM ethanol conditions, and these phenotypes were more obvious under high concentration of ethanol, whereas the tolerant accession Col-0 maintained more green seedlings as the ethanol concentration increased to 40 mM (Figure 4F,G). Upon desubmergence, high levels of ADH1 activity convert ethanol to toxic acetaldehyde [49,58]. Next, we analyzed both accessions seedlings germinated on solid MS medium with 5 mM acetaldehyde for two weeks, and we found that both accessions were sensitive to acetaldehyde, but L*er* seedlings were hypersensitive to acetaldehyde without root (Figure S8). These data indicate that accession L*er* displaying sensitivity to submergence and ethanol treatment may be resulted from the accumulation of toxicant acetaldehyde due to its higher ADH1 activity.

Taken together, these results suggest that the responses to ethanol and submergence may be connected.

### 2.7. Ler Plants Show Reduced Autophagy in Response to Submergence

**Figure 5:**
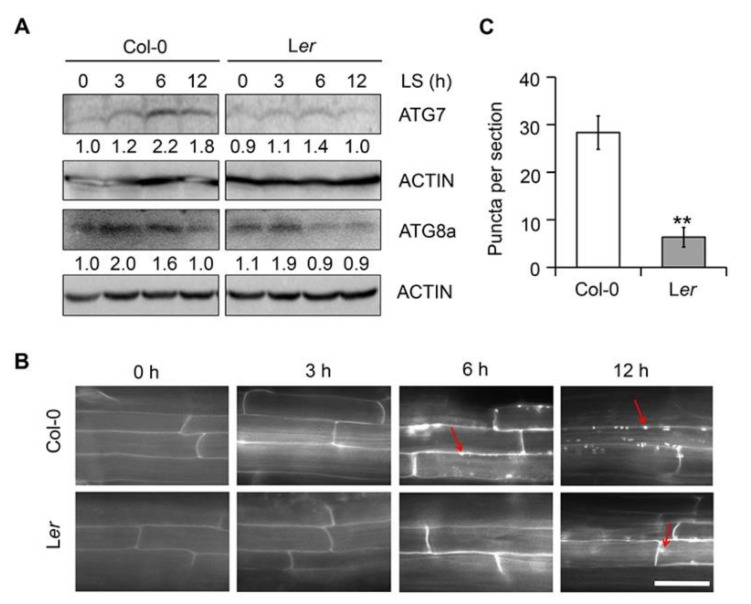
Autophagosome formation in two Arabidopsis accessions in response to submergence. (**A**) Protein abundance of ATG7 and ATG8a in one-week-old Col-0 and L*er* seedlings treated with light submergence (LS) at various time points. Numbers below the protein bands indicate relative gray values of the bands. Actin bands are shown below the blots to indicate the amount of protein loaded per lane. The experiments were repeated three times with similar results. (**B**) MDC staining of root cells from one-week-old Col-0 and L*er* seedlings treated with light submergence (LS) at various time points. Red arrows indicate labeled autophagosomes. The experiments were repeated three times with similar results. Bars = 50 µm. (**C**) Number of puncta per root section in mature root cells of one-week-old Col-0 and L*er* seedlings following LS treatment for 12 h in (**B**). Data are average values ± SD of three biological replicates. For each experiment, 15 sections were analyzed per accession. Asterisks indicate significant differences between Col-0 and L*er*, as determined by Student’s *t*-test (** *p* < 0.01).

The finding that ethanol is involved in autophagy-mediated submergence responses (Figure 1H and Figure 2) and that the levels of submergence tolerance of Arabidopsis accessions Col-0 and L*er* are similar to their tolerance to ethanol (Figure 4) raised the possibility that autophagy in response to submergence differs between the two Arabidopsis accessions. To test this hypothesis, we submerged 7-day-old Col-0 and L*er* seedlings for different periods of time and measured ATG7 and ATG8a levels by immunoblotting. As shown in Figure 5A, ATG7 levels in Col-0 seedlings gradually increased upon submergence and reached a maximum at 6 h, followed by a marked decrease after 12 h of submergence treatment. By contrast, in L*er* seedlings, ATG7 levels slightly increased in response to submergence and were significantly lower than that of Col-0 (Figure 5A). In both accessions, ATG8a levels increased in response to submergence and reached a peak at 6 h, which was followed by a decrease; this decrease was more rapid in L*er* than in Col-0 (Figure 5A).

To further confirm these findings, we observed autophagosome formation in the root cells of 7-day-old Col-0 and L*er* seedlings under submergence using MDC staining. As shown in Figure 5B, autophagosome formation was markedly induced in the root cells of Col-0 seedlings in response to 6 h and 12 h of submergence treatment; however, L*er* seedlings did not show autophagosomes formation until 12 h of treatment. The number of autophagosomes in response to submergence was significantly lower in L*er* seedlings than in Col-0 seedlings (Figure 5B,C).

Together, these observations indicate that submergence-induced autophagy is reduced in the Arabidopsis accession L*er*, leading to lower tolerance to submergence.

## 3. Discussion

We previously demonstrated that autophagy plays a pivotal role in regulating plant responses to submergence, which is an abiotic stress resulting in hypoxia and anaerobic respiration in plant cells [29,31,59]. In the present study, we obtained several lines of evidence that ethanol generated by anaerobic fermentation promotes autophagy, thereby increasing plant tolerance to submergence in Arabidopsis. First, transcriptome analysis identified numerous DEGs under ethanol treatment. In particular, the expression levels of genes involved in ROS metabolism and autophagy-related genes (*ATGs*) were highly correlated with plant responses to ethanol treatment (Figure 1G,H). Second, exogenous ethanol treatment activated autophagy in *GFP-ATG8e* transgenic plants (Figure 2). Third, the deficient ethanol production in adh1 reduced plant tolerance to submergence and decreased submergence-induced autophagosome formation (Figure 3). Finally, compared to Col-0, L*er* displayed enhanced sensitivity to submergence and ethanol stress along with higher ADH1 activity (Figure 4). These findings indicate that ethanol generated by anaerobic fermentation is essential for autophagy-mediated submergence tolerance in Arabidopsis.

Using different life history strategies, Arabidopsis has adapted during the course of evolution to diverse locations worldwide, spanning a wide geographic range and environmental conditions [60]. Therefore, natural populations of Arabidopsis must have developed abundant variations to help the plants respond to a variety of environments, making diverse Arabidopsis accessions powerful tools for identify the underlying key genes and causal mechanisms [61]. Increasing studies on plant morphology, physiology, and development as well as stress responses have estimated the natural variation in transcription between various Arabidopsis accessions [60,62,63]. An analysis of the natural variation of root hydraulics among a broad set of Arabidopsis accessions provided important clues about how hydraulic regulation allows plants to adapt to salt stress [64]. In addition, the transcriptional regulation of aquaporins is highly conserved among five Arabidopsis accessions in response to drought stress [65]. Degenkolbe et al. [66] performed mass spectrometry-based analysis to survey the lipid profiles of 15 Arabidopsis accessions and determined that the relative abundance of several lipid species is strongly linked to freezing tolerance, leading to the identification of possible marker lipids for plant freezing tolerance.

Vashisht et al. [53] detected natural phenotypic variation in submergence tolerance among 86 Arabidopsis accessions, ranging from the submergence-hypersensitive accession Cvi-0 to the submergence-tolerant accession C24. The accessions Col-0 and L*er* are moderately tolerant and sensitive to complete submergence, respectively [53], as verified in the current study. A comprehensive comparison of the shoot and root transcriptomic adjustments of eight Arabidopsis accessions in response to submergence and starvation stress uncovered conserved, organ-specific, and genotype-specific responses between submergence-tolerant and intolerant accessions. Importantly, chloroplast-encoded photosynthesis and redox-related genes are significantly upregulated in the root-specific transcriptome [54]. In addition, differences in the transcriptional regulation of *Respiratory Burst Oxidase Homolog D* (*RbohD*), *Senescence-Associated Gene 113* (*SAG113*), and *Oresara1* (*ORE1*) were detected between Arabidopsis accessions Bay-0 and Lp2-6 upon submergence stress. The authors suggested that a regulatory module consisting of RbohD, SAG113, and ORE1 controls ROS homeostasis, stomatal aperture, and chlorophyll degradation during submergence recovery [67].

In the current study, we established that ADH1 levels and activity were higher in submergence-intolerant L*er* compared to submergence-tolerant Col-0 in response to submergence stress. Ismond et al. [49] showed that *adh1* mutants are hypersensitive to submergence due to the loss of ethanolic fermentation. However, the overexpression of *ADH1* and increase in ADH activity do not affect ethanol levels and flooding survival tolerance under hypoxic conditions compared to wild type [49]. Possibly, the ADH levels in Col-0 are already in excess in anoxic or hypoxic conditions, and the ADH levels above a threshold do not correlate with ability to survive anoxia/hypoxia [49,58]. However, the exact threshold of ADH1 activity contributing to plant hypoxia tolerance is still unknown. We propose that the ADH levels of submergence-intolerant accession L*er* may exceed the threshold. It is also worth noting that high levels of ADH1 activity may play a crucial role in the subsequent aerobic conversion of ethanol to toxic acetaldehyde, which is a highly reactive chemical effecting cellular damage by forming acetaldehydeprotein adducts [49,58]. Consistent with this notion, our previous findings suggest that deficiencies of autophagy in the *atg* mutants lead to the upregulation of *ADH1* and hypersensitiveness to submergence [29]. Therefore, high levels of ADH activity in L*er* may catalyze the conversion of accumulated alcohol to acetaldehyde, leading to cellular damage during submergence or post-submergence recovery. It is likely that reasonable alcohol dehydrogenase activity and moderate ethanol signaling help submergence-tolerant accessions survive submergence stress.

*ADH1* overexpressing plants show improved tolerance to dehydration, cold, and salt stress as well as increased resistance to pathogen infection [18], suggesting that ADH1-mediated anaerobic respiration plays an important role in plant stress tolerance. The expression of the *ADH1* gene is induced by several environmental factors, such as cold, osmotic stress, and wounding, but the induction of hypoxia/anoxia is the strongest [68]. In addition, ethanol production induced by hypoxia/anoxia is much higher than that of other stress treatments [68]. Together, we conclude that the hypersensitivity of L*er* to submergence may due to the reduced autophagy and the accumulation of toxic acetaldehyde.

During the few past decades, numerous studies have investigated the transcriptomic adjustments of plants in response to hypoxia, including low-oxygen stress, waterlogging, and submergence stress with or without darkness [36,37,69]. Despite the complexity of the low-oxygen response, the specific conditions used for treatment, and the different organs and species subjected to transcriptome analysis, genes identified as being responsive to hypoxia primarily overlap with genes in the glycolysis and fermentative pathways. Specifically, strong increases in the expression of genes associated with anaerobic metabolism, such as *PDC1*, *ADH1*, and *SUS1*, were observed across plant species in response to oxygen deprivation, soil flooding, and submergence [36,69]. In addition to core metabolic reconfigurations that improve substrate-level ATP production and NAD^+^ regeneration, many plants species show an upregulation of genes encoding enzymes associated with ROS networks [69]. Here, by systematically exploring sets of upregulated and downregulated DEGs in response to ethanol, we confirmed much of what is currently known about the common core set of genes that function in the response to hypoxia stress, including genes associated with TFs and hormonal signaling (Figure 1B–E, Figure S2,3). More importantly, autophagy-related genes were significantly upregulated in response to ethanol (Figure 1H). Furthermore, ethanol stimulated the accumulation of autophagosomes (Figure 2). Indeed, we previously revealed that autophagy-defective (*atg*) mutants are hypersensitive to ethanol treatment and show high ROS accumulation [29].

Many animal models and in vitro cell culture models have been developed to study the relationship between ethanol, autophagy, and diseases. In mice with ethanol-induced hepatotoxicity and steatosis, autophagy was induced by ethanol exposure, which is a process that depends on ethanol metabolism and ROS [70]. Several mechanisms might contribute to ethanol-induced autophagy in neurons, such as the induction of oxidative stress and endoplasmic reticulum stress by ethanol and the disruption of intracellular calcium homeostasis under ethanol treatment [71]. Two additional studies in mammals suggested that autophagy is triggered by ethanol, which could help alleviate sarcopenia, as well as ethanol neurotoxicity [72,73]. Several reviews summarize the role of autophagy in alcohol-induced diseases and explain the important roles of ethanol metabolism and ROS in the disease formation process [71,74,75].

**Figure 6:**
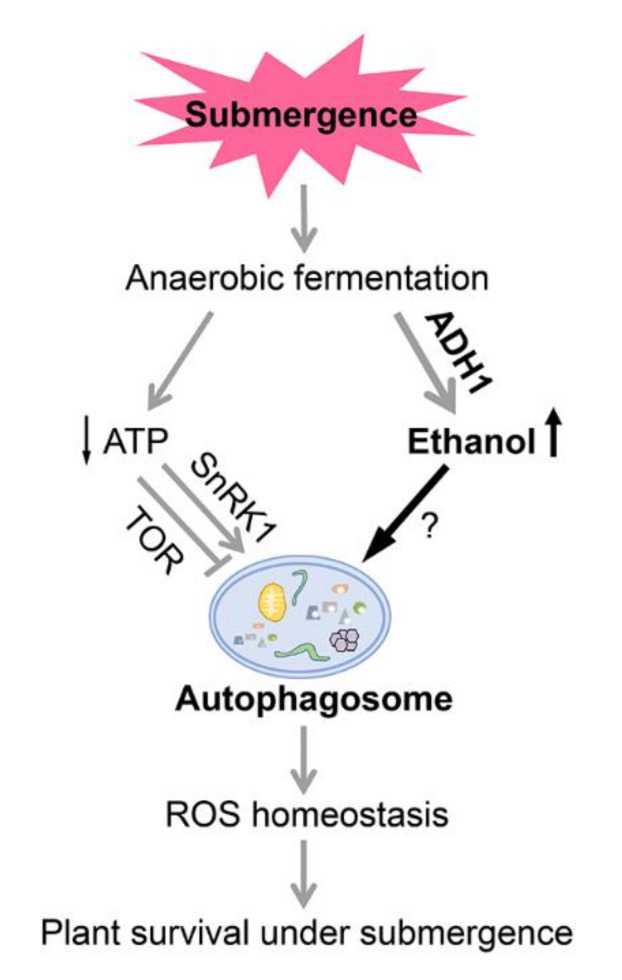
Proposed model for the role of ethanol-induced autophagy in regulating plant responses to submergence. Submergence causes hypoxia, which leads to deficiencies in cellular energy and carbohydrate shortages in plants. To survive hypoxic stress, plant cells switch from aerobic respiration to anaerobic fermentation, especially ethanolic fermentation. In this context, ADH1 (alcohol dehydrogenase1) activity increases rapidly in plant cells, resulting in the accumulation of ethanol. The increased ethanol levels promote autophagosome formation, which modulates hypoxia responses and facilitates plant survival by regulating ROS homeostasis, although the precise mechanism remains to be elucidated. By contrast, anaerobic fermentation produces much less ATP than aerobic respiration. The TOR (target of rapamycin) pathway and SnRK1 (Snf1-related protein kinase 1) play opposite roles in regulating autophagy in response to energy limitation. The energy sensor SnRK1 is activated under hypoxia stress and acts as a positive regulator of autophagy, whereas the negative effect of the TOR pathway on autophagy is repressed, ultimately improving plant survival following submergence.

Based on the current and previous findings, we propose a working model describing the role of ethanol-induced autophagy in regulating the response of Arabidopsis to submergence (Figure 6). According to our model, submergence causes hypoxia, which leads to deficiencies in cellular energy and carbohydrate shortages in plants. To survive hypoxic stress, plant cells switch from aerobic respiration to anaerobic fermentation, especially ethanolic fermentation. In this context, ADH1 activity increases rapidly in plant cells, resulting in the accumulation of ethanol. The increased ethanol levels help promote autophagosome formation to modulate hypoxia responses and facilitate plant survival by regulating ROS homeostasis, although the precise mechanism remains to be elucidated. On the other hand, much less ATP is produced by anaerobic fermentation compared to aerobic respiration. The TOR pathway and SnRK1 play opposite roles in inducing of autophagy in response to energy limitation. The energy sensor SnRK1 is activated by hypoxia stress and acts as a positive regulator of autophagy, whereas the negative effects of the TOR pathway on autophagy decrease under these conditions, ultimately improving plant survival following submergence. Further investigating the molecular basis for how anaerobic fermentation-generated ethanol activates autophagy will deepen our understanding of the upstream energy crisis that triggers the initiation of autophagy in plants.

## 4. Materials and Methods

### 4.1. Plant Material and Growth Conditions

Arabidopsis (*Arabidopsis thaliana*) accessions Columbia-0 (Col-0) and Landsberg *erecta* (L*er*) were used in this study. Mutants with insertions/deletions in the *ADH1* gene were obtained from the Arabidopsis Resource Center at Ohio State University (http://abrc.osu.edu) with the locus names *adh1-4* (CS66116), *adh1-8* (CS66117), and *adh1-16* (CS66118). Primers used for genotyping are listed in Table S5. The transgenic *GFP-ATG8e* lines were characterized as described previously [44]. Arabidopsis seeds were sterilized in 20% (*v/v*) chlorine bleach and 0.1% (*v/v*) Tween-20 for 10 min and washed five times with sterilize distilled water. Seeds were sown on Murashige and Skoog (MS) medium (Sigma-Aldrich) with 1% (*w/v*) sucrose and 0.8% (*w/v*) agar (pH 5.8) and stratified at 4 °C in the dark for 3 d. The plates were transferred to a growth room under a 16-h light/8-h dark (22 °C) photoperiod, and 7-day-old seedlings were transplanted to soil for further growth under the same conditions.

### 4.2. Plant Treatments

Submergence treatment was performed as described previously [50,76]. Briefly, 4-week-old plants were submerged at a depth of 10 cm beneath the water surface in the light (60 μmol m^−2^ s^−1^, normal light/dark cycle condition, LS) or constant darkness (DS), followed by a 6-day recovery under normal growth conditions. For DS treatment, control plants were placed in the dark in the same growth room. Plant samples were collected or photographed at the indicated time points. At least 15 plants per genotype were scored for dry weight following the 6-day recovery period. To calculate dry weight, the above-ground plant parts were harvested and heated overnight at 105 °C.

For ethanol or acetaldehyde treatment, sterilized seeds were sown on MS solid medium containing different concentrations of ethanol or acetaldehyde. Seedlings were scored and photographed at the indicated time points.

For immunoblotting analysis of GFP-ATG8e fusion protein, detached leaves from 4-week-old transgenic plants expressing *GFP-ATG8e* were immersed in 100 mM ethanol or water for 0, 3, 6, 12, and 24 h.

### 4.3. RNA Extraction and Gene Expression Analysis

Total RNA was isolated from the samples with TRIzol reagent (Invitrogen, USA), and cDNA was synthesized using a PrimeScript RT Reagent Kit with gDNA Eraser (Takara, China) according to the manufacturer’s instructions. RT-qPCR was performed using the StepOne Plus Real-time PCR System (Applied Biosystems) with SYBR Premix ExTaq Mix (Takara) as described previously [76,77]. The conditions for RT-qPCR were an initial step at 95 °C for 3 min, followed by 40 cycles of 10 s at 95 °C, 15 s at 55 °C, and 30 s at 72 °C. The RT-PCR program was 94 °C for 3 min, 25 cycles of 94 °C for 30 s, 55 °C for 30 s, 72 °C for 1 min, and 72 °C for 10 min. *ACTIN2* was used as the reference gene. The gene-specific primers for RT-qPCR and RT-PCR analysis are listed in Table S5.

### 4.4. Protein Extraction and Immunoblot Analysis

For total protein extraction, samples were ground in liquid nitrogen and homogenized in ice-cold extraction buffer (50 mM sodium phosphate (pH 7.0), 200 mM NaCl, 10 mM MgCl_2_, 0.2% (*v/v*) β-mercaptoethanol, 10% (*v/v*) glycerol) with protease inhibitor cocktail (Roche). To collect the supernatant for electrophoresis, extracts were incubated on ice for 30 min and centrifuged at 12,000× *g* for 30 min at 4 °C. For immunoblot analysis, total proteins were subjected to SDS-PAGE and electrophoretically transferred to nitrocellulose Western blotting membranes (Amersham) with a pore size of 0.2 µm or 0.45 µm PVDF (Immobilon, Merck, Germany). Immunoblot analysis was performed using anti-ADH1 (catalog no. AS10685, Agrisera, Sweden), anti-PDC1 (catalog no. AS10691, Agrisera, Sweden), anti-GFP (catalog no. 2955, CST, USA), anti-ATG7 (catalog no. ab53255, Abcam, UK), anti-ATG8a (catalog no. ab77003, Abcam, UK), and anti-ACTIN (catalog no. 58169, CST, USA) as the primary antibodies and horseradish peroxidase-conjugated anti-rabbit lgG (catalog no. A21020, Abbkine, China) and anti-mouse lgG (catalog no. A21010, Abbkine, China) as the secondary antibodies.

### 4.5. ADH Activity Measurements

ADH enzyme activity measurements were carried out as previously described [78] with minor modifications. Briefly, 2-week-old Arabidopsis seedlings were submerged in the dark and harvested at the indicated time points. The samples were homogenized in ice-cold extraction buffer (100 mM Tris–HCl, pH 8.0, 25% (*v/v*) glycerol, 0.8% (*v/v*) β-mercaptoethanol, 2% (*w/v*) polyvinylpyrrolidone, 5 mM dithiothreitol) and centrifuged at 13,000× *g* for 20 min at 4 °C. Protein concentrations were determined by Bradford assay. Fifty micrograms of protein were diluted in extraction buffer to a final volume of 150 µL. The enzymatic reaction was started by adding 800 µL activation buffer (62.5 mM Tris–HCl (pH 8.5), 1 mM NAD^+^), followed by 50 µL ethanol. Enzyme activity was determined based on the increase in absorbance at 340 nm every 30 s for 5 min.

### 4.6. RNA Sequencing Analysis

Transcriptome analysis was carried out according to Xie et al. [50]. Seven-day-old Col-0 seedlings were transferred to fresh MS agar medium for an additional 7 days of growth. Two-week-old seedings were drenched in 50 mM EtOH or water (control check, CK). After 12 h of treatment, seedlings were harvested. Each sample included three biological replicates, and each replicate included at least 60 seedlings. Total RNA was extracted from the samples using an RNeasy Plant Mini Kit (Qiagen) following the manufacturer’s instructions. Affymetrix ATH1 Arabidopsis chips (Affymetrix) were used for labeling, hybridization, scanning, and detection as described previously [79]. Affymetrix Gene Chip software MAS 5.0 and GeneSpring 12.6 (Agilent, USA) were used for raw data collection, normalization, and differentially expressed gene (DEG) identification using the criteria of fold change (FC) > 1.5-fold and *P* < 0.05. The R language was used for calculations and to generate plots.

### 4.7. Microscopy Analysis

GFP fluorescence in detached 4-week-old rosette leaves of *GFP-ATG8e* [44] was observed following treatment with 100 mM ethanol for 6 h; water treatment was used as a control (Mock). An LSM 780 NLO laser-scanning confocal microscope (Carl Zeiss, Germany) was used to observe the signals from GFP-ATG8e fusion protein. GFP fluorescence was detected at 488 nm filtered through a primary dichroic filter (UV/488/543).

MDC staining was carried out as described previously [29]. Briefly, 7-day-old seedlings were treated with or without light submergence (LS) in a 15-mL centrifuge tube containing 10 mL of sterile water for the indicated time and submersed in PBS buffer (135 mM NaCl, 4.7 mM KCl, 10 mM Na_2_HPO_4_, 2 mM NaH_2_PO_4_ [pH 7.4]) plus 0.05 mM monodansylcadaverine (MDC, Sigma-Aldrich) for 10 min. Following two rinses with PBS buffer, MDC-stained mature root cells were observed and photographed under an Axio Observer Z1 Inverted microscope (Carl Zeiss) using the 405 nm laser line.

## Supplementary Materials

Supplementary materials can be found at https://www.mdpi.com/1422-0067/21/19/7361/s1.

## Abbreviations

ABA	Abscisic acid
ADH	Alcohol dehydrogenase
AlaAT	Alanine aminotransferase
ATG7	AUTOPHAGY-RELATED PROTEIN 7
ATG8e	AUTOPHAGY-RELATED PROTEIN 8e
BR	Brassinosteroid
CK	Control check
DEGs	Differentially expressed genes
EtOH	Ethyl alcohol
FC	Fold change
FDR	False discovery rate
GA	Gibberellin
GFP	GREEN FLUORESCENT PROTEIN
JA	Jasmonic acid
LDH	Lactate dehydrogenase
MDC	Monodansylcadaverine
MS	Murashige and Skoog
PDC	Pyruvate decarboxylase
ROS	Reactive oxygen species
RT-PCR	Reverse transcription-polymerase chain reaction
RT-qPCR	Reverse transcription quantitative PCR
SA	Salicylic acid
SnRK1	Snf1-related protein kinase 1
TF	Transcription factor
TOR	Target of rapamycin
