# Supplementary material for: The Anaerobic Product Ethanol Promotes Autophagy-Dependent Submergence Tolerance in Arabidopsis
Source: Int J Mol Sci. 2020 Oct 5;21(19):7361. doi: 10.3390/ijms21197361 (PMC7583018; doi:10.3390/ijms21197361)
Supplement: Supplementary file 1 [file ijms-21-07361-s001.pdf]

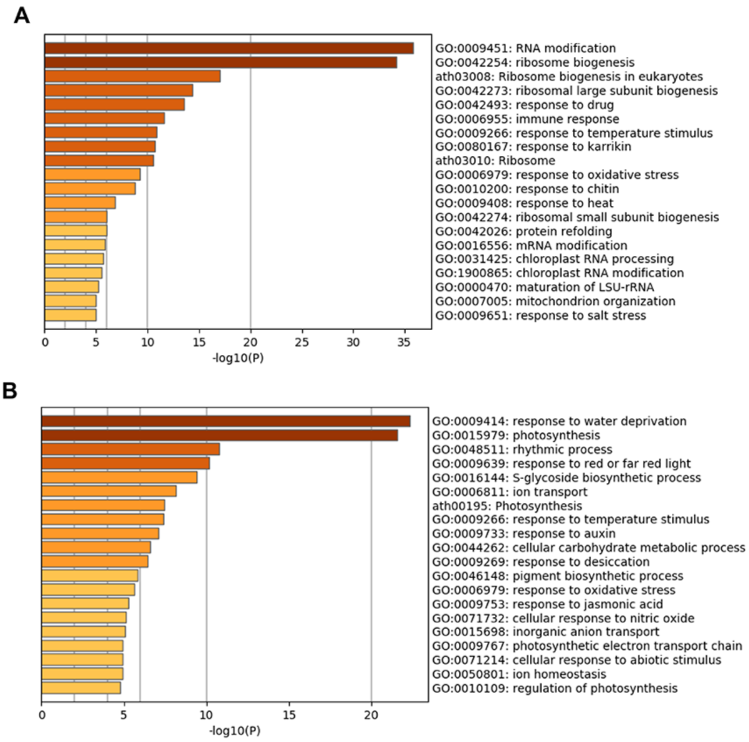

Figure S1. Enriched GO terms of DEGs in response to ethanol treatment.(**A**) Enriched GO terms of the 2,487 upregulated genes. Fold changes (FC) were calculated by comparing ethanol treatment to the control (0 h). Genes were identified as differentially expressed at FDR ≤ 0.005.

(**B**) Enriched GO terms of the 1,422 downregulated genes. Fold changes (FC) were calculated by comparing ethanol treatment to the control (0 h). Genes were identified as differentially expressed at FDR ≤ 0.005.

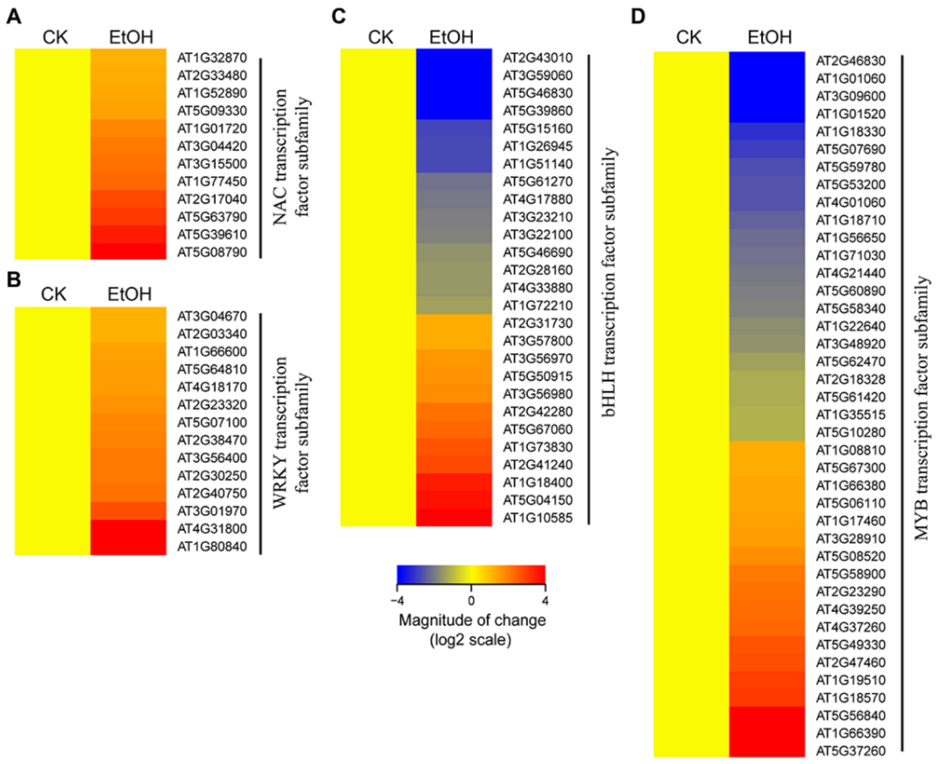

Figure S2. Differentially expressed transcription factor (TF) genes in response to ethanol treatment.
A to **D**: Hierarchical cluster analysis of the 12 differentially expressed NAC (**A**), 14 WRKY (**B**), 27 bHLH (**C**), and 40 MYB (**D**) transcription factor genes in response to ethanol treatment.
The log2 fold change values of the transcriptional profiles were calculated using the R program heatmap 2.0. Red and blue represent up- and downregulated genes, respectively. Differentially expressed genes were identified based on FDR < 0.005 and FC $\geqslant$ 1.5 or FC $\leqslant$ 0.67.

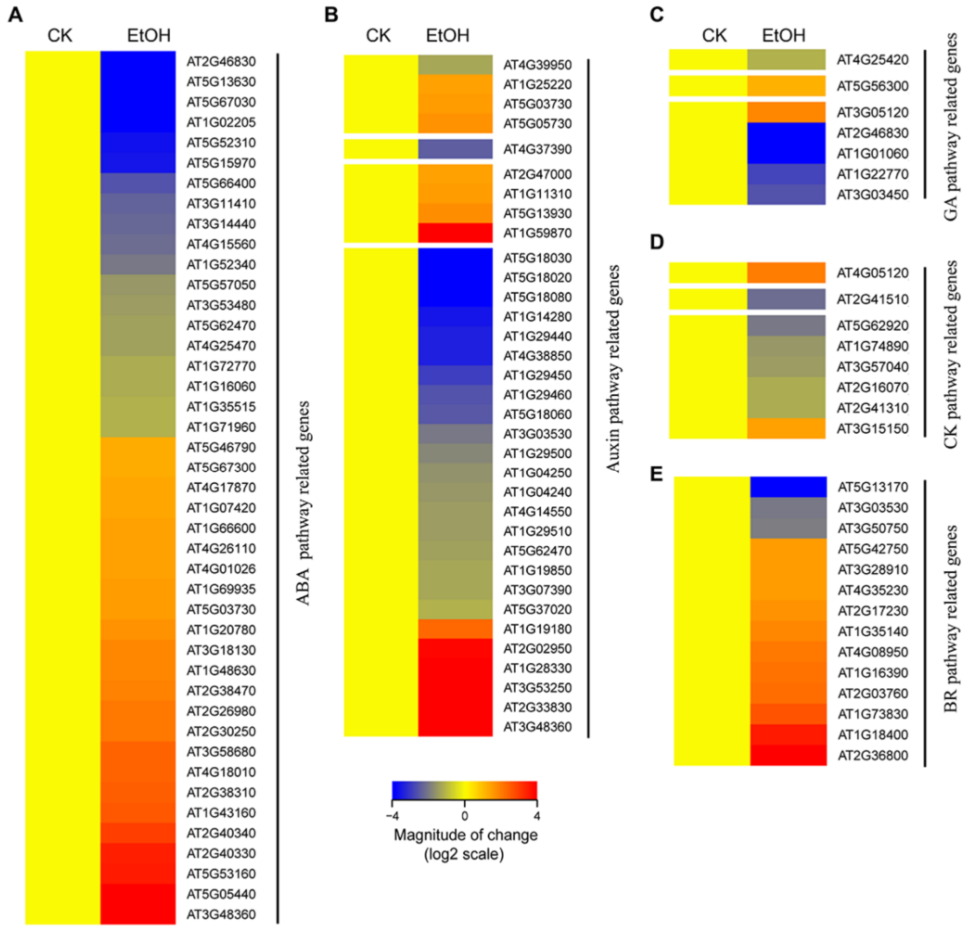

Figure S3. Differentially expressed phytohormone-related genes in response to ethanol treatment.

A to **E**: Hierarchical clustering of differentially expressed ethanol-responsive genes in the abscisic acid (ABA, **A**), auxin (**B**), gibberellin (GA, **C**), cytokinin (CK, **D**), and brassinosteroid (BR, **E**) biosynthesis and signaling pathways.

The log2 fold change values of the transcriptional profiles were calculated using the R program heatmap 2.0. Red and blue represent up- and downregulated genes, respectively. Differentially expressed genes were identified based on FDR < 0.005 and FC $\geqslant$ 1.5 or FC $\leqslant$ 0.67.

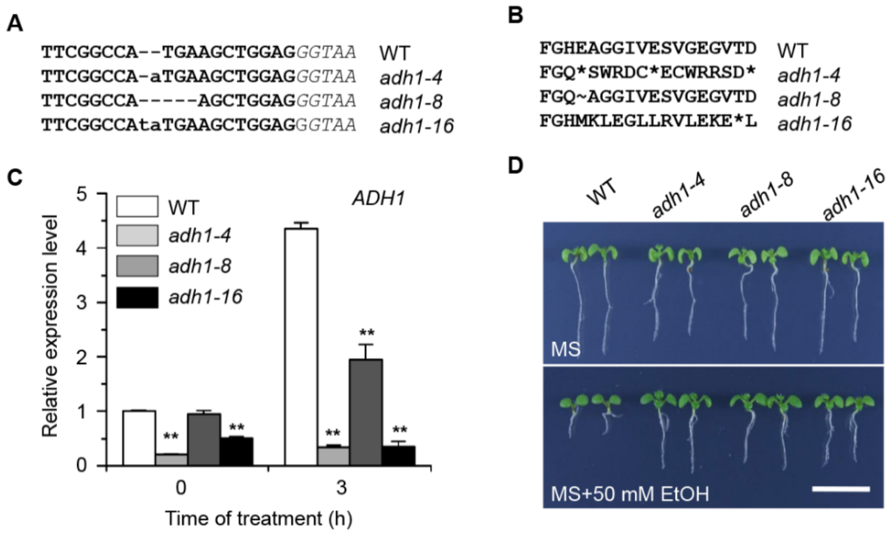

Figure S4. Characterization of *adh1* mutants. (**A**) Sequencing analysis showing the 1- and 2-bp insertions (indicated by lower-case letters) in the *adh1-4* and *adh1-16* mutants and the 3-bp deletion (indicated by hyphens) in the *adh1-8* mutant. (**B**) Predicted amino acid (aa) sequences showing premature translational termination (indicated by asterisks) in *adh1-4* and *adh1-16* and the 2-aa (H and E) deletions plus 1-aa introduction in the *adh1-8* mutant. (**C**) RT-qPCR analysis of the expression levels of *ADH1* in the wild type (WT) and *adh1* mutants (*adh1-4*, *adh1-8*, and *adh1-16*) upon light submergence (LS) treatment. Four-week-old plants were treated with LS for 0 and 3 h, and leaves were collected for RNA extraction. The mRNA abundance in WT at 0 h (using *ACTIN2* as the internal control) was set to 1. Data are average values ± SD of three technical replicates. Asterisks represent significant differences between WT and *adh* samples, as determined by Student's *t*-test (**$P < 0.01$). (**D**) Phenotypes of wild-type (WT), *adh1-4*, *adh1-8*, and *adh1-16* seedlings germinated on solid MS medium containing 0 or 50 mM ethanol and grown for 2 weeks. Bars = 1 cm. Experiments were performed three times with similar results.

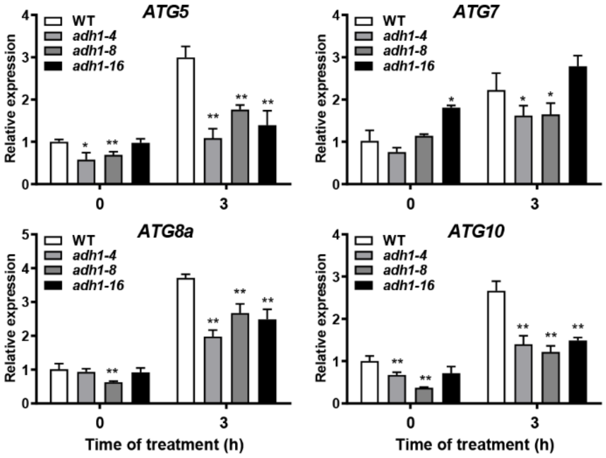

Figure S5. Relative expression levels of *ATG* genes in WT (Col-0) and *adh1* mutants (*adh1-4*, *adh1-8*, and *adh1-16*) upon light submergence (LS) treatment. Total RNA was isolated from 4-week-old WT and *adh1* mutant plants under LS treatment for 0 and 3 h, and leaves were collected for RNA extraction. The mRNA abundance in WT at 0 h (using *ACTIN2* as the internal control) was set to 1. Data are average values ± SD of three technical replicates. Asterisks represent significant differences between WT and *adh1* samples, as determined by Student's *t*-test (*$P < 0.05$; **$P < 0.01$). Experiments were performed three times with similar results.

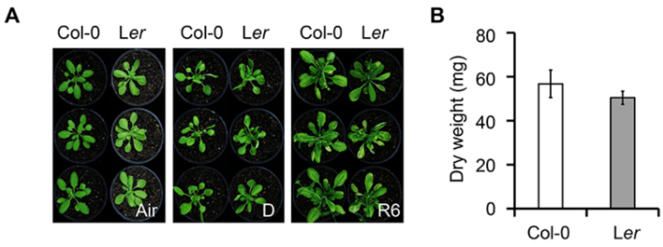

Figure S6. The phenotypes and dry weights of Col-0 and L*er* plants in response to dark treatment and recovery.（**A**）Phenotypes of 4-week-old Col-0 and L*er* plants before treatment (Air) and after 60 h dark treatment (D) with no submergence, followed by recovery for 6 d (R6).（**B**）Dry weights of Col-0 and L*er* plants with dark treatment followed by recovery for 6 d in (**A**). Data are means (±SD) of three biological replicates. For each biological replicate, 15 plants were analyzed per genotype.

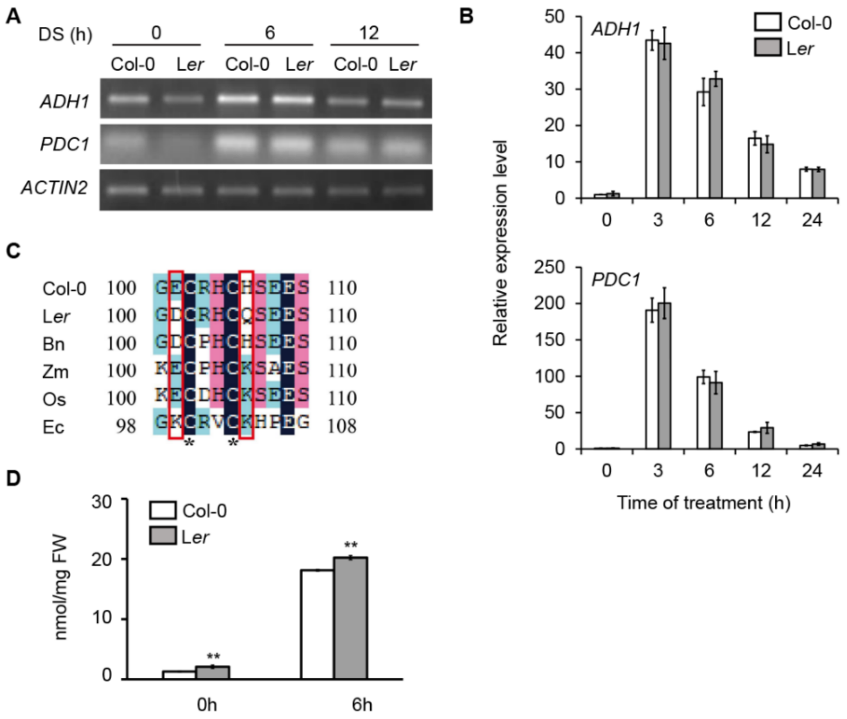

Figure S7. Characterization of *ADH1* and *PDC1* expression levels, amino acid sequences of ADH, and endogenous ethanol accumulation in accessions Col-0 and L*er*. (**A**) Analysis of *ADH1* and *PDC1* expression in response to DS treatment by RT-PCR.

Total RNA was isolated from 4-week-old Col-0 and L*er* plants treated with dark submergence (DS) for 0, 6, and 12 h. *ACTIN2* was amplified as an internal control. Experiments were performed three times with similar results. (**B**) Analysis of *ADH1* and *PDC1* expression in response to dark submergence (DS) by RT-qPCR. Total RNA was isolated from 4-week-old Col-0 and L*er* plants treated with DS treatment for 0, 3, 6, 12, and 24 h. The mRNA abundance in Col-0 at 0 h (using *ACTIN2* as the internal control) was set to 1. Experiments were performed three times with similar results. (**C**) Partial comparison of the amino acid sequences in the catalytic domain of ADH proteins from different species. Partial amino acid sequence alignments of ADH proteins are from Col-0, L*er*, rape (*Brassica napus*, Bn), maize (*Zea mays*, Zm), rice (*Oryza sativa*, Os) and horse liver (*Equus caballus*, Ec). The asterisks indicate Zn^{2+} binding sites in the catalytic domain of ADH proteins. Red boxes indicate the differences of ADH protein sequence. The Genbank accessions for rape, maize, rice and horse liver enzymes are AGB57581.1, AAB59302.1, BAE00046.1 and NP_001075414.1. (**D**) Endogenous ethanol accumulation in Col-0 and L*er*. Ethanol was extracted from 3-week-old plants untreated or immersed in 1% ethanol for 6 h. Four replicates were performed for each accession under each treatment. Values are mean ± SD and Student *t*-test was used to determine significant differences between the both accessions (**p<0.01).

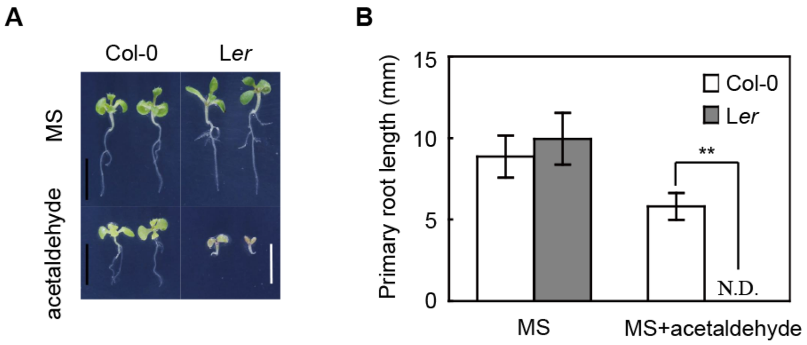

Figure S8. The phenotypes and primary root length of Col-0 and L*er* plants in response to acetaldehyde. (**A**) Phenotypes of Col-0 and L*er* on acetaldehyde plates. Both two accessions seeds were germinated on solid MS medium containing with or without 5 mM acetaldehyde and grown for 2 weeks. Bar = 5 mm. (**B**) Primary root length of Col-0 and L*er* in (**A**). Data are means (± SD) of three biological replicates. For each biological replicate, 14 plants were analyzed per accession. Asterisks represent significant differences between accession Col-0 and L*er* with 5 mM acetaldehyde treatment, as determined by Student's *t*-test (**$P < 0.01$). N.D means not detectable.
